# Influence of Growth Phase, pH, and Temperature on the Abundance and Composition of Tetraether Lipids in the Thermoacidophile *Picrophilus torridus*

**DOI:** 10.3389/fmicb.2016.01323

**Published:** 2016-08-30

**Authors:** Jayme Feyhl-Buska, Yufei Chen, Chengling Jia, Jin-Xiang Wang, Chuanlun L. Zhang, Eric S. Boyd

**Affiliations:** ^1^Department of Microbiology and Immunology, Montana State UniversityBozeman, MT, USA; ^2^State Key Laboratory of Marine Geology, Tongji UniversityShanghai, China; ^3^NASA Astrobiology InstituteMountain View, CA, USA

**Keywords:** tetraether, GDGT, GTGT, growth phase, temperature, pH, thermoacidophile, stress

## Abstract

The abundance and composition of glycerol dibiphytanyl glycerol tetraether (GDGT) and glycerol tribiphytanyl glycerol tetraether (GTGT) lipids were determined as a function of growth phase as a proxy for nutrient availability, the pH of growth medium, and incubation temperature in cultures of the thermoacidophile *Picrophilus torridus*. Regardless of the cultivation condition, the abundance of GDGTs and GTGTs was greater in the polar than core fraction, with a marked decrease in core GDGTs in cultures harvested during log phase growth. These data are consistent with previous suggestions indicating that core GDGTs are re-functionalized during polar lipid synthesis. Under all conditions examined, polar lipids were enriched in a GDGT with 2 cyclopentyl rings (GDGT-2), indicating GDGT-2 is the preferred lipid in this taxon. However, lag or stationary phase grown cells or cells subjected to pH or thermal stress were enriched in GDGTs with 4, 5, or 6 rings and depleted in GDGTs with 1, 2, 3, rings relative to log phase cells grown under optimal conditions. Variation in the composition of polar GDGT lipids in cells harvested during various growth phases tended to be greater than in cells cultivated over a pH range of 0.3–1.1 and a temperature range of 53–63°C. These results suggest that the growth phase, the pH of growth medium, and incubation temperature are all important factors that shape the composition of tetraether lipids in *Picrophilus*. The similarity in enrichment of GDGTs with more rings in cultures undergoing nutrient, pH, and thermal stress points to GDGT cyclization as a generalized physiological response to stress in this taxon.

## Introduction

Archaea are now known to populate nearly every niche inhabited by Bacteria (Cavicchioli, 2011). In addition, disproportionate abundances of Archaea relative to Bacteria have been reported in a number of “extreme” environments, including those characterized by elevated salt, temperature, and acidity (Valentine, 2007). It has previously been suggested that the prevalence of Archaea in such extreme environments is made possible by biomolecular adaptations that enable survival under conditions of chronic energy stress, including those made to the lipid membrane (Baker-Austin and Dopson, 2007; Valentine, 2007).

**Figure 1:**
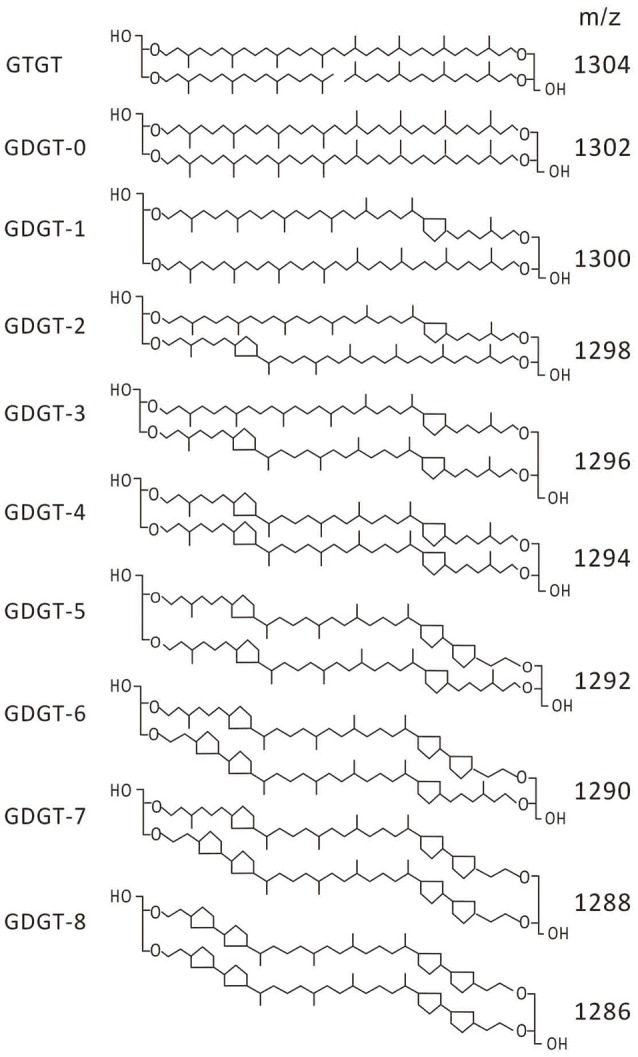
**Structures of GTGT and GDGT lipids identified in the membranes of *Picrophilus torridus***.

Archaea synthesize a variety of membrane lipids including glycerol dibiphytanyl glycerol tetraethers (GDGTs; De Rosa et al., 1980, 1986; De Rosa and Gambacorta, 1988; Macalady et al., 2004) which consist of two ether-linked C_40_ polyisoprenoid chains with zero to as many as four cyclopentyl rings and zero or one cyclohexyl ring [i.e., crenarchaeol (Sinninghe Damsté et al., 2002)] on each chain (Schouten et al., 2000, 2003; Figure 1). Glycerol tribiphytanyl glycerol tetraethers (GTGTs), which consist of a single ether-linked C_40_ polyisoprenoid chain with two ether-linked C_20_ polyisoprenoid chains (Figure 1), have also been detected in archaeal membranes, although they were in low abundance (Gulik et al., 1988; Hopmans et al., 2000; De La Torre et al., 2008; Pitcher et al., 2010, 2011; Elling et al., 2014). GTGTs have previously been proposed as an intermediate in the biosynthesis of a GDGT that contains zero cyclopentyl rings (i.e., GDGT-0) from two C_40_ diether core lipids (i.e., archaeol; Koga and Morii, 2007) while other authors have proposed GTGTs as a terminal biosynthetic product (Villanueva et al., 2014).

Once synthesized, the monolayer arrangement and the ether-linked bonding in GDGT lipids are thought to confer enhanced thermal stability (Thompson et al., 1992) and increased resistance to proton permeation (Yamauchi et al., 1993; Komatsu and Chong, 1998; Mathai et al., 2001). Likewise, the internal cyclopentyl rings are thought to increase the packing density of the lipid, thereby increasing the thermal stability of the membrane (Gliozzi et al., 1983; Gabriel and Chong, 2000) and further decreasing its permeability to protons. Despite the strong influence of temperature and pH on the composition of archaeal tetraether lipids observed in pure culture studies (De Rosa et al., 1980; Macalady et al., 2004; Boyd et al., 2011; Jensen et al., 2015), these parameters often fail to explain observed variation in the composition of tetraether lipids sampled from natural systems, in particular hydrothermal environments (Schouten et al., 2007; Pearson et al., 2008; Boyd et al., 2013; Jia et al., 2014). For example, less than 14% of the total variance in the composition of core or polar GDGT lipids sampled from hot springs in Yellowstone National Park (YNP), Wyoming could be accounted for by variation of temperature or pH (Boyd et al., 2013). A separate study of core GDGT lipids sampled from a variety of globally distributed hot springs found that both temperature and pH influenced the composition of archaeal lipids (Pearson et al., 2008); however, neither of these two parameters were capable of explaining the majority of the variation in the composition of GDGTs. Observations such as these suggest that other unaccounted for factors influence the abundance and composition of archaeal tetraether lipids in the natural environment.

One such factor that is likely to influence the abundance and composition of lipids in the natural environment is the growth state of the cells, which can be expected to be heterogeneous and to vary due to temporal or spatial variation in the availability of nutrients, toxic metabolic by-products, or other geochemical parameters (Roszak and Colwell, 1987; Morita, 1993; Kovárová-Kovar and Egli, 1998; Finkel, 2006). Indeed, studies of numerous bacterial taxa have shown the composition of membrane lipids to vary markedly as a function of growth phase (Marr and Ingraham, 1962; Veerkamp, 1971; Annous et al., 1999; Syakti et al., 2006). For example, *Escherichia coli* cells harvested from stationary phase cultures had a larger percentage of cyclopropane fatty acids (methylene hexadecanoic and methylene octadecanoic acids) when compared to cells harvested during exponential growth (Marr and Ingraham, 1962). Moreover, *E. coli* cells grown in chemostats under glucose limitation had a higher abundance of unsaturated fatty acids whereas cells grown under fixed nitrogen limitation had a higher abundance of saturated fatty acids, when compared to batch cultures (Marr and Ingraham, 1962). More recently, the composition of membrane lipids of the euryarchaeote *Methanobacter thermoautotrophicus* was shown to be modulated in response to nutrient limitation, with distinct differences in the composition of those lipids depending on whether cells were starved for phosphate and potassium or for hydrogen (Yoshinaga et al., 2015). Under phosphate limited conditions, cells produced a greater proportion of sodiated adducts of archaeol, whereas under hydrogen limited conditions cells produced a greater proportion of monoglycosidic and core archaeol lipids.

Additional insights into the influence of nutrient availability on the abundance and composition of lipids in archaea comes from a study of the lipids of the mesophilic, marine thaumarchaeote *Nitrosopumilus maritimus* as a function of growth phase (Elling et al., 2014), a proxy for nutrient availability. Actively growing cells (with adequate nutrients) of *N. maritimus* had a greater abundance of polar GDGTs when compared to cells harvested during stationary phase (nutrient limitation or waste product buildup); no change in the abundance of core GDGTs per cell was noted over the growth cycle. In a separate study it was shown that O_2_ limitation resulted in enrichment of GDGTs with 2 or 3 cyclopentyl rings in four marine thaumarchaeote isolates at the expense of GDGT-1 (Qin et al., 2015). Similarly, the abundance of intact polar lipids was shown to vary in *Thermococcus kodakarensis* cells harvested during exponential vs. stationary phase, in particular in cells that were grown with limited amounts of organic carbon or phosphate (Meador et al., 2014). Moreover, it was shown that the composition of GDGTs differed substantially in *N. maritimus* and *T. kodakarensis* cells harvested during various phases of growth (Elling et al., 2014; Meador et al., 2014). The average number of cyclopentyl rings decreased in cultures of the thermophilic crenarchaeotes *Sulfolobus islandicus and S. tokadaii* harvested at mid log phase, when compared to lag and stationary phase cultures regardless of the incubation temperature (Jensen et al., 2015). Together, these results strongly suggest that nutrient limitation, either imposed directly by limiting the supply of nutrients at the start of an experiment or indirectly by sampling cells over a growth period as nutrients become limiting due to microbial activity, strongly influence archaeal membrane lipid composition. Moreover, these results may indicate that stress imposed by nutrient limitation influences the membrane lipid composition to an equal or greater degree than stress imposed by suboptimal incubation temperature.

While previous studies have compared the relative influence of growth phase (proxy for nutrient limitation) and suboptimal temperature on tetraether lipid composition (Jensen et al., 2015), to our knowledge, no studies have compared the relative influence of growth phase and suboptimal pH on tetraether lipid composition, despite the strong effect that pH apparently has on archaeal lipid composition (Boyd et al., 2011). The thermoacidophilic euryarchaeotes *Picrophilus torridus* and *P. oshimae* (Thermoplasmatales), which grow optimally at 58–60°C and at a pH of 0.7 (Schleper et al., 1995, 1996), represent the most acid tolerant acidophilic organisms known to date. The lipids of *P. oshimae* comprise primarily GDGTs (Schleper et al., 1995) and liposomes prepared from *P. oshimae* extracts are extremely impermeable to protons (Van De Vossenberg et al., 1998), consistent with the perceived role of GDGTs in adaptation to low pH (Yamauchi et al., 1993; Komatsu and Chong, 1998; Mathai et al., 2001; Macalady et al., 2004; Boyd et al., 2011, 2013). Unlike most archaea, *P. torridus and P. oshimae* grow to high densities (Schleper et al., 1995, 1996) which makes them suitable strains for lipidomic studies. In the present study we examined the lipidomes of cells of *P. torridus* as a function of growth phase, the pH of the growth medium, and incubation temperature. These data were then used to evaluate the relative influence of nutrient stress (i.e., growth phase) and stress imposed by suboptimal pH and temperature on the abundance and composition of GDGTs.

## Materials and methods

### Growth conditions

A culture of *Picrophilus torridus* DSM 9790, originally isolated from an acidic geothermally heated solfatara in Northern Japan (Schleper et al., 1995), was obtained from the American Type Culture Collection (ATCC 700027). Cells were grown on ATCC 2011 medium which consists of (NH_4_)_2_SO_4_ (0.2 g L^−1^), MgSO_4_ (0.5 g L^−1^), CaCl_2_ • 2H_2_O (0.25 g L^−1^), KH_2_PO_4_ (3.0 g L^−1^), and yeast extract (2.0 g L^−1^). Unless otherwise stated, the pH of the medium was adjusted to 0.7 with concentrated H_2_SO_4_. Growth medium (250 mL) was dispensed into 500 mL Erlenmeyer flasks and was autoclave sterilized at 121°C for 21 min. Cells were grown on a MaxQ model 4450 (ThermoScientific, Waltham, MA) shaking incubator (75 rpm) at a temperature of 58°C, unless otherwise stated. Growth was monitored optically at an absorbance at 600 nm and by direct cell counting using a Petroff-Hauser counting chamber (Hausser Scientific, Horsham, PA) following manufacturer protocols. Light microscopy was also used to qualitatively evaluate cell size. It is possible that cell size varied during growth conditions and was not noticed via our analyses made using light microscopy. However, the fact that a significant relationship was observed between measurements of growth using optical methods (absorbance at 600 nm) and by direct cell counting (Pearson *R*^2^ = 0.99; Supplementary Figure 1) suggests that differences in cell size during growth under variable conditions are minimal.

Growth curves based on optical density and cell number were obtained for triplicate cultures grown at optimal pH and temperature (pH 0.7, 58°C), as well as in cultures grown at 58°C with the medium pH adjusted to 0.3, 0.5, 0.9, and 1.1 (Supplementary Figures 2A,B). Growth curves were also obtained in cultures grown in medium with a pH of 0.7 incubated at 53 and 63°C (Supplementary Figure 2C). Generation times under varied pH conditions, when calculated using absorbance at 600 nm (Supplementary Figure 3), were similar to those reported previously (Schleper et al., 1995). However, when calculated using direct cell counts, generation times were slightly shorter than reported previously (Supplementary Figure 3). Under optimal growth conditions, cultures were harvested at time points corresponding to lag phase, early log phase, log phase, late log phase, and early stationary phase (Supplementary Figure 2A). Under all other growth conditions (pH and temperature series), cultures were harvested during log phase. Cells were harvested by centrifugation (10,000 x g, 15 min, 4°C). Cell pellets from cultures containing a defined number of cells were frozen at −80°C and were freeze dried for use in lipid extraction.

### Tetraether lipid separation, detection, and quantification

Freeze-dried cells were homogenized with a mortar and pestle before lipid extraction. Homogenized cells were extracted following a modified Bligh and Dyer extraction procedure (Sturt et al., 2004). Samples were sequentially extracted five times by sonication for 10 min. using a mixture of methanol (MeOH), dichloromethane (DCM), and phosphate buffer (PB; pH 7.4) (2:1:0.8, v/v/v). After each sonication, samples were centrifuged at 2500 rpm for 2 min, and were then subjected to the next round of extraction. Equal volumes of Milli-Q water and DCM were added to adjust the ratio of MeOH:DCM:PB to 1:1:0.9 (v/v/v) and to achieve phase separation. The bottom layer of these extractions was collected and subjected to this process another two times, without the addition of Milli-Q water. Milli-Q water was again added to the final volume and mixed vigorously before being placed at 4°C for 30 min. Following this settling period, the supernatant was removed and the sample dried under nitrogen gas. Samples were re-suspended through the addition of a small volume of ethyl acetate:*n*-hexane (1:1, v/v) and sonication. Core lipids were obtained via elution with *n*-hexane and ethyl acetate (1:1, v/v) in a silica gel column constructed of extracted silica suspended in a mixture of *n*-hexane and ethyl acetate (1:1, v/v). Following this elution, MeOH (100%) was added to the column to elute polar lipids (Tierney et al., 2012). Prior to drying under nitrogen, 111.7 ng of C_46_ GTGT standard was added as an internal standard to each collection of polar or core lipids for each sample (Huguet et al., 2006). One half of the polar fraction was analyzed directly and the other half was hydrolyzed according to previously described methods through the addition of MeOH and HCl (95:5, v/v) and sonication prior to heating at 70°C for 3 h (Wei et al., 2011). Thus, polar lipids are defined as what is released by acid hydrolysis (hydrolysable GDGTs), which was corrected for the amount of core lipids present. Following heating, the bottom layer was sequentially extracted four times through the addition of equal volumes of DCM and Milli-Q water and the sample dried under nitrogen. All samples were re-dissolved and sonicated in *n*-hexane:isopropanol (99:1, v/v) and were filtered through 0.45 μm PTFE filters prior to drying under nitrogen, resuspension in *n*-hexane:isopropanol (99:1, v/v), and injection on the liquid chromatograph.

GDGTs were analyzed on an Agilent 1200 liquid chromatograph equipped with an automatic injector coupled to triple quadrapole (QQQ) 6460 MS loaded with Mass Hunter software according to previously defined procedures (Zhang et al., 2011). Separation of peaks was achieved using a Prevail Cyano column (2.1 × 150 mm, 3 μm; Alltech Deerfield, IL) maintained at a temperature of 40°C. Injection volume was 10 μL. Two solvents were used in the elution of GDGTs, solvent A (*n*-hexane) and solvent B (90% *n*-hexane: 10% isopropanol). GDGTs were first eluted isocratically with 90% solvent A and 10% solvent B for 5 min., followed by a linear gradient to 18% solvent B in 45 min. The solute was held for 10 min. in 100% solvent B and was then allowed to re-equilibrate in a mixture of solvents A:B (9:1, v/v) for 10 min.

Detection of GDGTs was performed using QQQ mass spectrometry with an atmospheric pressure chemical ionization ion source. The scanning type used was the single ion monitoring mode of protonated molecules. The conditions for APCI/MS were as follows: nebulizer pressure 40 psi, vaporizer temperature 350°C, drying gas (N_2_) flow 5L min^−1^ and temperature 250°C, capillary voltage 3 kV, and corona 4 μA. All samples were quantified by correlating peak areas of the samples to those derived by adding a known amount of an internal C_46_ standard. Prior to lipid extraction, powdered samples were weighed in order to allow for normalization of lipid concentrations from the resulting lipid extracts to previously determined cell numbers in each sample. GDGTs identified by LC-MS are reported according to the nomenclature of (Schouten et al., 2003) and as modified in (Pearson et al., 2008). The weighted average number of GDGT rings per lipid molecule [Ring Index (RI)] was calculated according to the formula: RI = [%GDGT-1 + 2^*^(%GDGT-2) + 3^*^(%GDGT-3) + 4^*^(%GDGT-4) + 5^*^(%GDGT-5) + 6^*^(%GDGT-6) + 7^*^(%GDGT-7) + 8^*^(%GDGT-8)]/100 as modified from Schouten et al. (2007).

## Results

### Influence of growth phase on tetraether lipid abundance and composition

**Figure 2:**
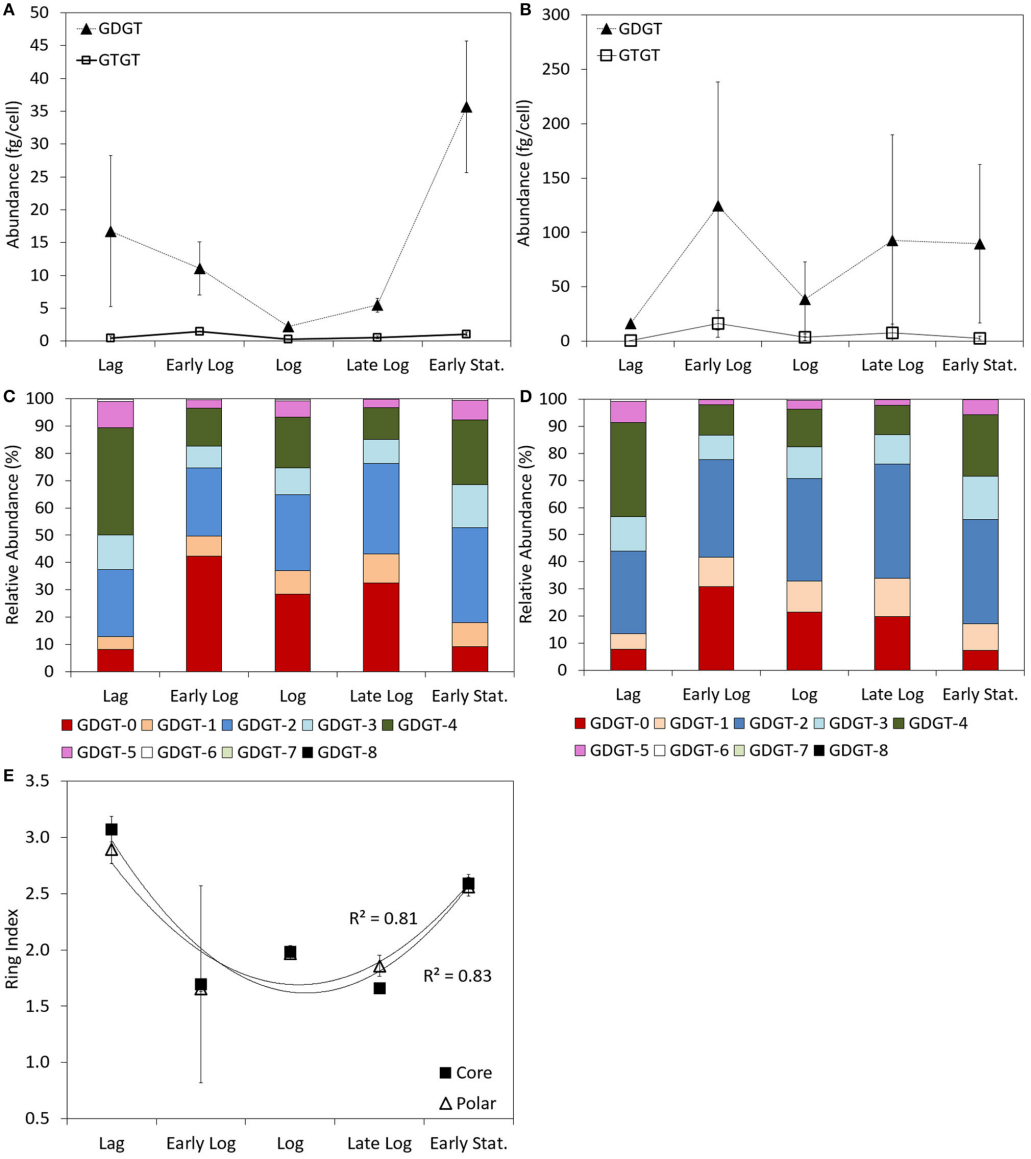
**Abundance of GDGT and GTGTs per cell in the core (A) and polar (B) fractions of batch cultures of *P. torridus* sampled during various phases of growth**. Relative abundance of individual GDGT lipids in the core **(C)** and polar **(D)** lipid fractions of cells of *P. torridus* harvested at different phases of growth. Calculated ring indices for cultures of *P. torridus* harvested at varying stages of growth **(E)**. All cultures were grown in medium with a pH of 0.7 and were incubated at 58°C. Error bars in panels **(A,B,E)** represent the standard deviation of three replicate cultures.

Both GDGTs and GTGTs were detected in the core and the polar lipid fractions of *P. torridus* cells harvested at all stages of growth when incubated at 58°C in medium with a pH of 0.7. In the core fraction, the abundance of GDGTs was greater than the abundance of GTGTs regardless of growth phase. The abundance of core GDGTs ranged from 2.2 ± 0.3 to 35.6 ± 10.0 fg cell^−1^ during the growth cycle (Supplementary Table 1), with the highest abundance per cell detected during lag and early stationary phase grown cultures and the lowest abundance detected during log phase growth (Figure 2A). The abundance of GTGTs in the core fraction ranged from 0.3 ± < 0.1 to 1.4 ± < 0.1 fg cell^−1^ over this same growth cycle (Supplementary Table 1) but, unlike the abundance of core GDGTs, did not vary systematically as a function of growth phase (Figure 2A).

Similar to the core fraction, the abundance of GDGTs was greater than GTGTs in the polar fraction regardless of growth phase. In the polar fraction, the abundance of GDGTs ranged from 16.3 ± 0.3 to 124.3 ± 114.1 fg cell^−1^ during the growth cycle (Supplementary Table 1) and their abundance did not vary systematically during the growth cycle (Figure 2B). The abundance of GTGTs in the polar fraction ranged from 0.6 ± < 0.1 to 16.0 ± 12.3 fg cell^−1^ over this same growth cycle (Supplementary Table 1) and also did not vary as a function of growth phase (Figure 2B).

GDGTs containing between 0 and 8 cyclopentyl rings per molecule were observed in the core and polar fraction of *P. torridus* cells grown under optimal conditions in all phases of growth (Supplementary Table 1, Figures 2C,D); however, the relative abundance of individual lipids varied. Core GDGTs in cells harvested during lag phase and early stationary phase cells primarily consisted of GDGT-2, -3, -4, and -5 (Figure 2C). This difference was reflected in variation in the core GDGT ring index over the growth cycle, which was greater in cells harvested during lag and early stationary phase than in cells harvested during early log, log, and late log phase (Supplementary Table 1; Figure 2E). In contrast, during early log, log, and late log phase growth, core GDGTs primarily consisted of GDGT-0 with a reduced abundance of GDGT-3, -4, and -5. Similar to the core fraction, the predominant polar GDGTs in cells harvested during lag and stationary phases of growth were comprised primarily of GDGT-2, -3, -4, and -5 (Supplementary Table 1; Figure 2D). When compared to lag and early stationary phase cultures, cultures harvested during early log, log, and late log phase growth exhibited an increase in polar GDGT-0, -1 and a reduction in GDGT-3, -4, and -5. Like the core lipid fraction, this difference was reflected in variation in the polar GDGT ring index over the growth cycle, which was greater in cells harvested during lag and early stationary phase than in cells harvested during early log, log, and late log phase (Supplementary Table 1; Figure 2E).

### Influence of pH on tetraether lipid abundance and composition

**Figure 3:**
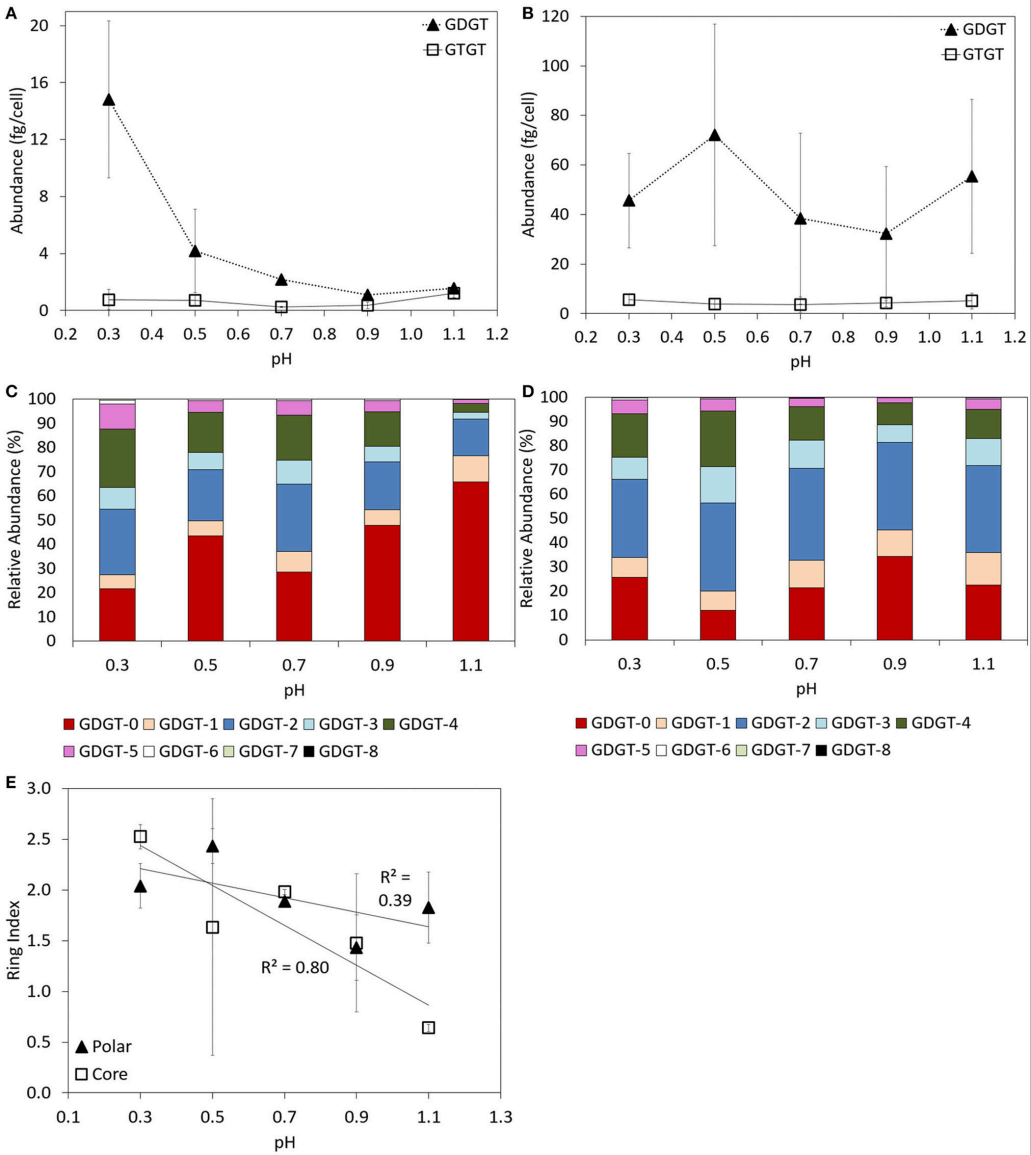
**Abundance of GDGT and GTGTs per cell in the core (A) and polar (B) fractions of batch cultures of *P. torridus* grown in cultivation medium with varying pH**. Relative abundance of individual GDGT lipids in the core **(C)** and polar **(D)** lipid fractions of log phase cells of *P. torridus* grown in cultivation medium with varying pH. Calculated ring indices for log phase cultures of *P. torridus* grown in cultivation medium with varying pH **(E)**. All cultures were incubated at 58°C. Error bars in panels **(A,B,E)** represent the standard deviation of three replicate cultures.

Both GDGTs and GTGTs were identified in the core and polar lipid fraction of log phase cells of *P. torridus* incubated at 58°C when grown in media with an initial pH of 0.3, 0.5, 0.7, 0.9, and 1.1 (Supplementary Table 2). In the core fraction, GDGTs were always more abundant than GTGTs (Figure 3A). The abundance of core GDGTs per cell decreased with increasing pH of the cultivation medium, with the highest abundance of core GDGTs detected in cells grown at pH 0.3 (14.8 ± 5.5 fg cell^−1^) and the lowest in cells grown at pH 0.9 (1.1 ± 0.1 fg cell^−1^). Systematic trends were not observed in the abundance of polar GDGTs or GTGTs as a function of the pH of the growth medium (Figure 3B). However, the abundance of GDGTs and GTGTs per cell was greater in the polar fraction than in the core fraction, regardless of the pH of the cultivation medium (Supplementary Table 2).

GDGTs containing between 0 and 8 cyclopentyl rings per molecule were observed in the core and polar fraction of log phase *P. torridus* cells incubated at 58°C in cultivation medium with the pH adjusted to values ranging from 0.3 to 1.1 (Supplementary Table 2, Figures 3C,D); however, the relative abundance of individual lipids varied. In the core fraction, the relative abundance of GDGTs-2, -4, -5, -6 tended to be higher in cells grown at more acidic pH, whereas GDGT-0 and GDGT-1 were more abundant in cells grown at higher pH, which was reflected in an inverse correlation (Pearson *R*^2^ = 0.80) between the core GDGT ring index and pH (Figure 3D). Variations in the relative abundance of individual GDGTs in the polar fraction were less apparent. GDGT-1 was less abundant in cells grown at more acidic pH, and the number of cyclopentyl rings per GDGT (i.e., ring index) varied inversely (Pearson *R*^2^ = 0.39) with the pH of the cultivation medium (Figure 3E).

### Influence of incubation temperature on tetraether lipid abundance and composition

**Figure 4:**
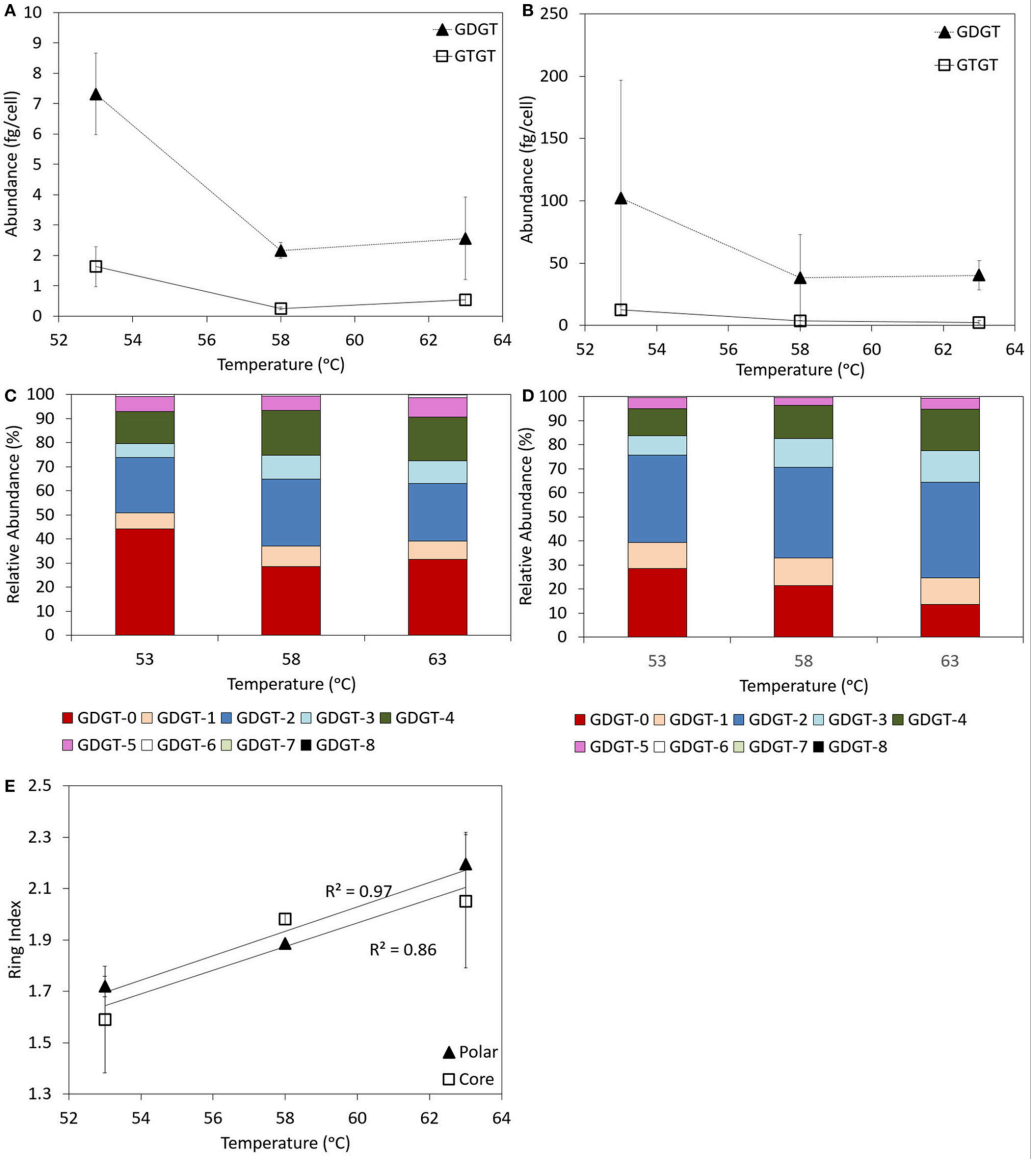
**Abundance of GDGT and GTGTs per cell in the core (A) and polar (B) fractions of batch cultures of *P. torridus* grown at different incubation temperatures**. Relative abundance of individual GDGT lipids in the core **(C)** and polar **(D)** lipid fractions of log phase cells of *P. torridus* incubated at different temperatures. Calculated ring indices for log phase cultures of *P. torridus* grown at varying incubation temperatures **(E)**. All cultures were grown in media with a pH of 0.7. Error bars in panels **(A,B,E)** represent the standard deviation of three replicate cultures.

Both GDGTs and GTGTs were identified in the core and polar lipid fraction of log phase cells of *P. torridus* grown in cultivation medium with a pH of 0.7 and incubated at 53, 58, and 63°C (Supplementary Table 3). In both the core and polar fractions, GDGTs were always more abundant than GTGTs, with the abundance of core and polar GDGTs per cell decreasing with increasing incubation temperature (Figures 4A,B).

GDGTs containing between 0 and 8 cyclopentyl rings per molecule were observed in the core and polar fraction of log phase *P. torridus* cells grown in cultivation medium with a pH of 0.7 when incubated at 53, 58, and 63°C (Supplementary Table 3, Figures 4C,D). In the core fraction, the relative abundance of GDGTs did not vary systematically in cultures incubated at 58 and 63°C. However, substantial differences were apparent in cultures incubated at 53°C, in particular the higher relative abundance of GDGT-0. The core GDGT ring index was positively correlated (Pearson *R*^2^ = 0.86) with temperature (Figure 4E), and this relationship was primarily driven by variation in GDGTs associated with cultures incubated at 53°C. In the polar fraction, the abundance of GDGT-3 and GDGT-4 increased with increasing incubation temperature, whereas the abundance of GDGT-0 decreased with increasing incubation temperature. As such, the polar GDGT ring index was positively correlated (Pearson *R*^2^ = 0.97) with incubation temperature (Figure 4E).

### Comparison of the influence of growth phase, pH of cultivation medium, and incubation temperature on tetraether lipid abundance and composition

The abundance of polar GDGTs and GTGTs per cell across all growth treatments was 60.4 ± 32.5 fg cell^−1^ and 5.5 ± 4.3 fg cell^−1^, respectively, whereas the abundance of core GDGTs and GTGTs per cell across all growth treatments was 8.2 ± 9.7 fg cell^−1^ and 0.7 ± 0.4 fg cell^−1^, respectively (Supplementary Tables 1–3). The highest abundance of polar GDGTs and GTGTs were from early log phase cells grown under optimal conditions or in cells grown at a lowered temperature of 53°C. The highest abundance of core GDGTs was observed in lag and early stationary phase cells grown under optimal conditions and in cells grown in increasingly acidic medium. The abundance of core GTGTs did not vary markedly in any of the growth conditions tested.

Core and polar GDGTs with 0, 2, or 4 cyclopentyl rings were the most abundant components in the cell membranes regardless of culture conditions (Supplementary Tables 1–3). The Bray Curtis (BC) index of similarity was used to compare the net variation in polar and core GDGT lipid profiles across all cultivation conditions. The BC similarity in polar GDGT individual lipid profiles was the lowest when comparing lag and early log phase harvested cells or lag and late log phase harvested cells (BC similarity = 0.66 and 0.68, respectively; Table 1). The BC similarity in core GDGT individual lipid profiles was the lowest (0.51) in cells cultivated at pH 0.3 when compared to pH 1.1, and was also low (0.61) in a comparison between lag and late-log phase cells grown under optimal conditions.

**Table 1:** **Matrix describing the Bray-Curtis similarity in the composition of GDGT lipids in the core and polar fraction of cells harvested at various points during their growth cycle (pH 0.7, 58°C), in log phase cells grown at 58°C in medium with varying pH, or in log phase cells grown in medium with a pH of 0.7 at varying incubation temperatures**.

**Core**	**Lag**	**Early Log**	**Log**	**Late Log**	**Early Stat**.	**pH 0.3**	**pH 0.5**	**pH 0.7**	**pH 0.9**	**pH 1.1**	**53°C**	**58°C**	**63°C**
Lag	**1.00**	0.63	0.72	0.61	0.82	0.81	0.63	0.72	0.59	0.36	0.62	0.72	0.74
Early Log	0.63	**1.00**	0.86	0.88	0.67	0.78	0.94	0.86	0.92	0.73	0.94	0.86	0.88
Log	0.72	0.86	**1.00**	0.89	0.81	0.89	0.85	1.00	0.81	0.60	0.84	1.00	0.94
Late Log	0.61	0.88	0.89	**1.00**	0.75	0.78	0.82	0.89	0.80	0.67	0.83	0.89	0.87
Early Stat.	0.82	0.67	0.81	0.75	**1.00**	0.83	0.66	0.81	0.61	0.41	0.65	0.81	0.76
pH 0.3	0.81	0.78	0.89	0.78	0.83	**1.00**	0.78	0.89	0.73	0.51	0.77	0.89	0.88
pH 0.5	0.63	0.94	0.85	0.82	0.66	0.78	**1.00**	0.85	0.95	0.73	0.95	0.85	0.88
pH 0.7	0.72	0.86	1.00	0.89	0.81	0.89	0.85	**1.00**	0.81	0.60	0.84	1.00	0.94
pH 0.9	0.59	0.92	0.81	0.80	0.61	0.73	0.95	0.81	**1.00**	0.78	0.95	0.81	0.83
pH 1.1	0.36	0.73	0.60	0.67	0.41	0.51	0.73	0.60	0.78	**1.00**	0.74	0.60	0.62
53°C	0.62	0.94	0.84	0.83	0.65	0.77	0.95	0.84	0.95	0.74	**1.00**	0.84	0.87
58°C	0.72	0.86	1.00	0.89	0.81	0.89	0.85	1.00	0.81	0.60	0.84	**1.00**	0.94
63°C	0.74	0.88	0.94	0.87	0.76	0.88	0.88	0.94	0.83	0.62	0.87	0.94	**1.00**
**Polar**	**Lag**	**Early Log**	**Log**	**Late Log**	**Early Stat**.	**pH 0.3**	**pH 0.5**	**pH 0.7**	**pH 0.9**	**pH 1.1**	**53°C**	**58°C**	**63°C**
Lag	**1.00**	0.66	0.73	0.68	0.85	0.77	0.85	0.73	0.62	0.72	0.68	0.73	0.79
Early Log	0.66	**1.00**	0.90	0.89	0.75	0.89	0.78	0.90	0.96	0.92	0.97	0.90	0.83
Log	0.73	0.90	**1.00**	0.93	0.84	0.89	0.86	1.00	0.87	0.96	0.92	1.00	0.92
Late Log	0.68	0.89	0.93	**1.00**	0.79	0.82	0.80	0.93	0.85	0.93	0.88	0.93	0.88
Early Stat.	0.85	0.75	0.84	0.79	**1.00**	0.81	0.94	0.84	0.72	0.81	0.77	0.84	0.91
pH 0.3	0.77	0.89	0.89	0.82	0.81	**1.00**	0.85	0.89	0.85	0.89	0.91	0.89	0.86
pH 0.5	0.85	0.78	0.86	0.80	0.94	0.85	**1.00**	0.86	0.75	0.84	0.81	0.86	0.92
pH 0.7	0.73	0.90	1.00	0.93	0.84	0.89	0.86	**1.00**	0.87	0.96	0.92	1.00	0.92
pH 0.9	0.62	0.96	0.87	0.85	0.72	0.85	0.75	0.87	**1.00**	0.88	0.94	0.87	0.79
pH 1.1	0.72	0.92	0.96	0.93	0.81	0.89	0.84	0.96	0.88	**1.00**	0.94	0.96	0.89
53°C	0.68	0.97	0.92	0.88	0.77	0.91	0.81	0.92	0.94	0.94	**1.00**	0.92	0.85
58°C	0.73	0.90	1.00	0.93	0.84	0.89	0.86	1.00	0.87	0.96	0.92	**1.00**	0.92
63°C	0.79	0.83	0.92	0.88	0.91	0.86	0.92	0.92	0.79	0.89	0.85	0.92	**1.00**

*Bold-faced values correspond to identical comparisons while grayed areas highlight pairwise comparisons for lipidomes obtained from cells harvested during various phases of growth or cells grown in medium with differing pH or incubation temperatures*.

## Discussion

The abundance of GDGTs in the polar fraction of *P. torridus* averaged 60.4 ± 32.5 fg cell^−1^ and ranged from 16.5 to 124.3 fg cell^−1^, which is between 1 and 3 orders of magnitude higher than the 0.82–1.81 fg cell^−1^ determined for the marine thaumarchaeote *N. maritimus* (Elling et al., 2014). Elling et al. (2014) attributed variation in the total lipid content of cells of *N. maritimus* to differences in cell size, which can vary according to the growth state of cells. However, noticeable changes in the size of cells over the growth cycle were not noted in the present study using microscopic methods. Cells of *P. torridus* are coccoid (spherical) and have a diameter of ~1 μm (Schleper et al., 1996), which equates to a cell surface area of 3.14 μm^2^. In contrast *N. maritimus* cells are rod shaped (cylindrical) with width of 0.2 μm and a variable length ranging from 0.5 to 0.9 μm (Konneke et al., 2005), which equates to a cell surface area of 0.38–0.63 μm^2^, respectively. Since the primary lipids in *P. torridus* (Schleper et al., 1995) and *N. maritimus* (Schouten et al., 2008) are GDGTs with a small contribution of GTGTs, it is likely safe to assume that their membranes are of a similar thickness. To the extent that this is true, then the density of polar GDGT lipids per unit surface area in *P. torridus* cells (range of 5.25–39.49 fg per μm^2^) is slightly higher than for *N. maritimus* cells (range of 1.26–4.73 fg per μm^2^; Elling et al., 2014). Why the abundance of polar GDGTs in cells of *P. torridus* is higher than in cells of *N. maritimus* is not immediately clear. One possible explanation is that differences between studies are attributable to a correction imposed on data for response factors of purified standards by Elling et al. (2014), which was not done in the current study. Alternatively, it is possible that differences exist in the efficiency of lipid extraction between the two different organisms, which belong to different archaeal phyla.

The abundance of polar GDGTs did not vary systematically as a function of growth phase, which differs from *N. maritimus* cells which had a higher density of polar GDGTs per cell during active phases of growth (Elling et al., 2014). However, core GDGTs were depleted in cells harvested during log phase when compared to those harvested in lag or early stationary phase. This may suggest that core GDGTs are re-functionalized during synthesis of polar lipids in actively dividing (log phase) archaeal cells or may suggest that polar GDGTs are degraded to core GDGTs during cell death. These observations are consistent with previous interpretations of the distribution of core and polar GDGTs, which indicated that core GDGTs are recycled during cellular growth (Takano et al., 2010; Liu et al., 2011). In further support of this hypothesis is the observation that ring indices for core and polar GDGTs in cells harvested during various stages of growth and when cultivated over a range of temperatures and pH regimes were similar. The high degree of similarity in the composition of core and polar GDGTs, as suggested by the calculated ring index, indicates a dynamic relationship between the production and/or fate of core and polar GDGTs in *P. torridus*, a relationship that may extend to other archaeal taxa.

Culture and environmental studies have previously suggested that archaea increase the degree of lipid cyclization in response to increasing temperature (De Rosa et al., 1980; Uda et al., 2001; Shimada et al., 2008; Boyd et al., 2011; Jensen et al., 2015) and increasing acidity (Macalady et al., 2004; Pearson et al., 2008; Boyd et al., 2011, 2013). Indeed the average number of cyclopentyl rings per GDGT molecule (ring index) increased in the polar and core fraction of cells grown at progressively higher temperature (Figure 3E). In particular, cells grown at higher temperatures were enriched in GDGT-3 and -4 and depleted in GDGT-0. Likewise, the extent of cyclization of core GDGTs in cells of *P. torridus* increased with increasing acidity of the growth medium as indicated by an increase in the ring index. However, the extent of cyclization of polar GDGTs did not correlate as well with the acidity of the cultivation medium when compared to core GDGTs. It is possible that the increase in the ring index in the core fraction of cells grown at increasingly acidic pH is due to preferential re-functionalization of GDGTs using core GDGTs with few or no cyclopentyl rings (e.g., GDGT-0) as a substrate. This would lead to an apparent increase in the core GDGT ring index while not substantially affecting the polar GDGT ring index.

Enrichment of GDGTs with fewer cyclopentyl rings (GDGT-0, -1, -2) and depletion of GDGTs with a greater number of cyclopentyl rings (GDGT-3, -4, -5) resulted in a decreased ring index for log phase cells when compared to lag and early stationary phase cultures. This finding is consistent with Jensen et al. (2015) who showed a dip in the ring index in cells of *S. islandicus* and *S. tokodaii* harvested during log phase relative to those harvested during lag or stationary phase. These observations suggest that actively growing crenarcheotes (e.g., *S. islandicus* and *S. tokodaii*) and euryarcheotes (e.g., *P. torridus*) produce GDGTs with fewer cyclopentyl rings and that less active cells produce GDGTs with more rings. It is not clear from these data if addition of rings takes place after condensation of the two archaeol lipids (Koga and Morii, 2007) or if this takes place prior to condensation of archaeol lipids (Villanueva et al., 2014).

The growth state of cells in natural systems is likely to be heterogeneous due to temporal or spatial variation in the availability of nutrients as well as toxic metabolic by-products (Roszak and Colwell, 1987; Morita, 1993; Kovárová-Kovar and Egli, 1998; Finkel, 2006). In addition to nutrient availability, the growth state of cells can also be influenced by dynamic changes in the geochemical conditions of an environment, including thermal or pH fluctuations. Intriguingly, log phase *P. torridus* cells that were cultivated under thermal stress (i.e., incubation at 63°C) and to a lesser extent pH stress (i.e., grown in medium with a pH of 0.3) were also enriched in GDGT-3, -4, and -5 and depleted in GDGT-0 and -1 relative to log phase cells grown under optimal conditions. Thus, it is possible that the composition of GDGTs in archaeal cells is reflective of general physiological stress imposed by nutrient limitation or suboptimal growth conditions, which are likely related in that the latter growth conditions would increase the demand for nutrients (Shock and Holland, 2007). When considered in light of results showing the central role of the role of nutrient limitation (Matsuno et al., 2009; Meador et al., 2014; Jensen et al., 2015; Qin et al., 2015; Yoshinaga et al., 2015), thermal stress (De Rosa et al., 1980; Uda et al., 2001; Shimada et al., 2008; Boyd et al., 2011; Jensen et al., 2015), and pH stress (Boyd et al., 2011) in shaping archaeal lipid composition, these observations may help to reconcile why previous attempts to describe variation in archaeal lipids in natural environments are only marginally successful. This may be particularly true for highly dynamic environments, such as geothermal springs (Pearson et al., 2008; Boyd et al., 2013; Jia et al., 2014), which vary in temperature and chemistry on scales that range from minutes to decades (Hurwitz and Lowenstern, 2014). Such conditions can impose stress on cells, either due to fluctuating geochemistry or fluctuating nutrient availability. Alternatively, it has been suggested that archaeal phylogeny is an important determinant of GDGT lipid composition (Villanueva et al., 2014; Xie et al., 2014). If true, it follows that the relationship between environmental pH or temperature and GDGT lipid composition may be indirectly due to the constraints imposed by those variables on the assembly of geothermal communities (Alsop et al., 2014). The fact that a relationship exists between taxonomy, GDGT distribution, and environmental pH or temperature (Boyd et al., 2013) supports the central role for GDGTs in the habitation of acidic high temperature ecosystems (Valentine, 2007).

## Conclusions

Variation in lipid abundance and composition in *P. torridus* cells harvested during various growth phases tended to be greater or equal to that associated with cells cultivated over a pH range of 0.3–1.1 and a temperature range of 53–63°C. Enrichment of GDGT-3, -4, and -5 and depletion of GDGT-0 and -1 in less active cells relative to active cells and in cells undergoing pH or thermal stress relative to those grown under optimal conditions suggests that GDGT composition may be a physiological response to stress. Since growth phase is contingent on nutrient supply and the build-up of toxic metabolic byproducts, these results indicate an important role for nutrient stress in dictating the abundance and composition of tetraethers in *P. torridus*, and possibly other archaeal taxa. Collectively these data add to our understanding of the factors that influence the composition and abundance of tetraether lipids in taxonomically diverse archaea.

## Author contributions

JF grew and harvested the cultures. JF, YC, CJ, and JW extracted lipids and analyzed via mass spectrometry. CZ and EB oversaw the work and wrote the paper.

### Conflict of interest statement

The authors declare that the research was conducted in the absence of any commercial or financial relationships that could be construed as a potential conflict of interest.

## References

[B1] Alsop E. B. Boyd E. S. Raymond J. (2014). Merging metagenomics and geochemistry reveals environmental controls on biological diversity and evolution. BMC Ecol. 14:16. 10.1186/1472-6785-14-16 24886397 PMC4047435

[B2] Annous B. A. Kozempel M. F. Kurantz M. J. (1999). Changes in membrane fatty acid composition of *Pediococcus* sp. strain NRRL B-2354 in response to growth conditions and its effect on thermal resistance. Appl. Environ. Microbiol. 65, 2857–2862. 10388676 10.1128/aem.65.7.2857-2862.1999 PMC91429

[B3] Baker-Austin C. Dopson M. (2007). Life in acid: pH homeostasis in acidophiles. Trends Microbiol. 15, 165–171. 10.1016/j.tim.2007.02.005 17331729

[B4] Boyd E. S. Hamilton T. L. Wang J. He L. Zhang C. L. (2013). The role of tetraether lipid composition in the adaptation of thermophilic Archaea to acidity. Front. Microbiol. 4:62. 10.3389/fmicb.2013.00062 23565112 PMC3615187

[B5] Boyd E. S. Pearson A. Pi Y. Li W.-J. Zhang Y. He L. (2011). Temperature and pH controls on glycerol dibiphytanyl glycerol tetraether lipid composition in the hyperthermophilic crenarchaeon *Acidilobus sulfurireducens*. Extremophiles 15, 59–65. 10.1007/s00792-010-0339-y 21125411

[B6] Cavicchioli R. (2011). Archaea — timeline of the third domain. Nat. Rev. Microbiol. 9, 51–61. 10.1038/nrmicro2482 21132019

[B7] De La Torre J. R. Walker C. B. Ingalls A. E. Könneke M. Stahl D. A. (2008). Cultivation of a thermophilic ammonia oxidizing archaeon synthesizing crenarchaeol. Environ. Microbiol. 10, 810–818. 10.1111/j.1462-2920.2007.01506.x 18205821

[B8] De Rosa M. Esposito E. Gambacorta A. Nicolaus B. Bu'lock J. D. (1980). Effects of temperature on the lipid composition of *Caldariella acidophila*. Phytochemistry 19, 827–831.

[B9] De Rosa M. Gambacorta A. (1988). The lipids of archaebacteria. Prog. Lipid Res. 27, 153–175. 10.1016/0163-7827(88)90011-2 3151021

[B10] De Rosa M. Gambacorta A. Gliozzi A. (1986). Structure, biosynthesis, and physicochemical properties of archaebacterial lipids. Microbiol. Rev. 50, 70–80. 3083222 10.1128/mr.50.1.70-80.1986 PMC373054

[B11] Elling F. J. Könneke M. Lipp J. S. Becker K. W. Gagen E. J. Hinrichs K.-U. (2014). Effects of growth phase on the membrane lipid composition of the thaumarchaeon *Nitrosopumilus maritimus* and their implications for archaeal lipid distributions in the marine environment. Geochim. Cosmochim. Acta 141, 579–597. 10.1016/j.gca.2014.07.005

[B12] Finkel S. E. (2006). Long-term survival during stationary phase: evolution and the GASP phenotype. Nat. Rev. Microbiol. 4, 113–120. 10.1038/nrmicro1340 16415927

[B13] Gabriel J. L. Chong P. L. G. (2000). Molecular modeling of archaebacterial bipolar tetraether lipid membranes. Chem. Phys. Lipids 105, 193–200. 10.1016/S0009-3084(00)00126-2 10823467

[B14] Gliozzi A. Paoli G. De Rosa M. Gambacorta A. (1983). Effect of isoprenoid cyclization on the transition temperature of lipids in thermophilic archaebacteria. Biochim. Biophys. Acta 735, 234–242. 10.1016/0005-2736(83)90298-5

[B15] Gulik A. Luzzati V. Derosa M. Gambacorta A. (1988). Tetraether lipid components from a thermoacidophilic archaebacterium: chemical structure and physical polymorphism. J. Mol. Biol. 201, 429–435. 10.1016/0022-2836(88)90149-0 3138418

[B16] Hopmans E. C. Schouten S. Pancost R. D. Van Der Meer M. T. J. Sinninghe Damsté J. S. (2000). Analysis of intact tetraether lipids in archaeal cell material and sediments by high performance liquid chromatography/atmospheric pressure chemical ionization mass spectrometry. Rapid Comm. Mass Spectrom. 14, 585–589. 10.1002/(SICI)1097-0231(20000415)14:7<585::AID-RCM913>3.0.CO;2-N 10775092

[B17] Huguet C. Hopmans E. C. Febo-Ayala W. Thompson D. H. Sinninghe Damsté J. S. Schouten S. (2006). An improved method to determine the absolute abundance of glycerol dibiphytanyl glycerol tetraether lipids. Org. Geochem. 37, 1036–1041. 10.1016/j.orggeochem.2006.05.008

[B18] Hurwitz S. Lowenstern J. B. (2014). Dynamics of the Yellowstone hydrothermal system. Rev. Geophys. 52, 375–411. 10.1002/2014RG000452

[B19] Jensen S. M. Neesgaard V. L. Skjoldbjerg S. L. Brandl M. Ejsing C. S. Treusch A. H. (2015). The effects of temperature and growth phase on the lipidomes of *Sulfolobus islandicus* and *Sulfolobus tokodaii*. Life 5:1539. 10.3390/life5031539 26308060 PMC4598652

[B20] Jia C. Zhang C. L. Xie W. Wang J.-X. Li F. Wang S. (2014). Differential temperature and pH controls on the abundance and composition of H-GDGTs in terrestrial hot springs. Org. Geochem. 75, 109–121. 10.1016/j.orggeochem.2014.06.009

[B21] Koga Y. Morii H. (2007). Biosynthesis of ether-type polar lipids in Archaea and evolutionary considerations. Microbiol. Mol. Biol. Rev. 71, 97–120. 10.1128/MMBR.00033-06 17347520 PMC1847378

[B22] Komatsu H. Chong P. L.-G. (1998). Low permeability of liposomal membranes composed of bipolar tetraether lipids from thermoacidophilic archaebacterium *Sulfolobus acidocaldarius*. Biochemistry 37, 107–115. 10.1021/bi972163e 9425030

[B23] Könneke M. Bernhard A. E. De La Torre J. R. Walker C. B. Waterbury J. B. Stahl D. A. (2005). Isolation of an autotrophic ammonia-oxidizing marine archaeon. Nature 437, 543–546. 10.1038/nature03911 16177789

[B24] Kovárová-Kovar K. Egli T. (1998). Growth kinetics of suspended microbial cells: from single-substrate-controlled growth to mixed-substrate kinetics. Microbiol. Mol. Biol. Rev. 62, 646–666. 9729604 10.1128/mmbr.62.3.646-666.1998 PMC98929

[B25] Liu X. Lipp J. S. Hinrichs K.-U. (2011). Distribution of intact and core GDGTs in marine sediments. Org. Geochem. 42, 368–375. 10.1016/j.orggeochem.2011.02.003

[B26] Macalady J. L. Vestling M. M. Baumler D. Boekelheide N. Kasper C. W. Banfield J. F. (2004). Tetraether-linked membrane monolayers in *Ferroplasma* spp: a key to survival in acid. Extremophiles 8, 411–419. 10.1007/s00792-004-0404-5 15258835

[B27] Marr A. G. Ingraham J. L. (1962). Effect of temperature on the composition of fatty acids in *Escherichia coli*. J. Bacteriol. 84, 1260–1267. 16561982 10.1128/jb.84.6.1260-1267.1962 PMC278056

[B28] Mathai J. C. Sprott G. D. Zeidel M. L. (2001). Molecular mechanisms of water and solute transport across archaebacterial lipid membranes. J. Biol. Chem. 276, 27266–27271. 10.1074/jbc.M103265200 11373291

[B29] Matsuno Y. Sugai A. Higashibata H. Fukuda W. Ueda K. Uda I.. (2009). Effect of growth temperature and growth phase on the lipid composition of the archaeal membrane from *Thermococcus kodakaraensis*. Biosci. Biotechnol. Biochem. 73, 104–108. 10.1271/bbb.80520 19129645

[B30] Meador T. B. Gagen E. J. Loscar M. E. Goldhammer T. Yoshinaga M. Y. Wendt J.. (2014). *Thermococcus kodakarensis* modulates its polar membrane lipids and elemental composition according to growth stage and phosphate availability. Front. Microbiol. 5:10. 10.3389/fmicb.2014.00010 24523718 PMC3906577

[B31] Morita R. Y. (1993). Bioavailability of Energy and the Starvation State, in Starvation in Bacteria, ed Kjelleberg S. (New York, NY: Springer US), 23.

[B32] Pearson A. Pi Y. Zhao W. Li W. Li Y. Inskeep W. (2008). Factors controlling the distribution of archaeal tetraethers in terrestrial hot springs. Appl. Environ. Microbiol. 74, 3523–3532. 10.1128/AEM.02450-07 18390673 PMC2423032

[B33] Pitcher A. Hopmans E. C. Mosier A. C. Park S.-J. Rhee S.-K. Francis C. A.. (2011). Core and intact polar glycerol dibiphytanyl glycerol tetraether lipids of ammonia-oxidizing Archaea enriched from marine and estuarine sediments. Appl. Environ. Microbiol. 77, 3468–3477. 10.1128/AEM.02758-10 21441324 PMC3126447

[B34] Pitcher A. Rychlik N. Hopmans E. C. Spieck E. Rijpstra W. I. C. Ossebaar J.. (2010). Crenarchaeol dominates the membrane lipids of *Candidatus* Nitrososphaera gargensis, a thermophilic Group I.1b Archaeon. ISME J. 4, 542–552. 10.1038/ismej.2009.138 20033067

[B35] Qin W. Carlson L. T. Armbrust E. V. Devol A. H. Moffett J. W. Stahl D. A.. (2015). Confounding effects of oxygen and temperature on the TEX86 signature of marine Thaumarchaeota. Proc. Nat. Acad. Sci. U.S.A. 112, 10979–10984. 10.1073/pnas.1501568112 26283385 PMC4568219

[B36] Roszak D. B. Colwell R. R. (1987). Survival strategies of bacteria in the natural environment. Microbiol. Rev. 51, 365–379. 3312987 10.1128/mr.51.3.365-379.1987 PMC373117

[B37] Schleper C. Puehler G. Holz I. Gambacorta A. Janekovic D. Santarius U.. (1995). *Picrophilus* gen. nov., fam. nov.: a novel aerobic, heterotrophic, thermoacidophilic genus and family comprising archaea capable of growth around pH 0. J. Bacteriol. 177, 7050–7059. 8522509 10.1128/jb.177.24.7050-7059.1995 PMC177581

[B38] Schleper C. Puhler G. Klenk H.-P. Zillig W. (1996). *Picrophilus oshimae* and *Picrophilus torridus* fam. nov., gen. nov., sp. nov., two species of hyperacidophilic, thermophilic, heterotrophic, aerobic Archaea. Int. J. Syst. Bacteriol. 46, 814–816. 10.1099/00207713-46-3-814

[B39] Schouten S. Hopmans E. C. Baas M. Boumann H. Standfest S. Könneke M. (2008). Intact membrane lipids of “Candidatus *Nitrosopumilus maritimus*,” a cultivated representative of the cosmopolitan mesophilic Group I Crenarchaeota. Appl. Environ. Microbiol. 74, 2433–2440. 10.1128/AEM.01709-07 18296531 PMC2293165

[B40] Schouten S. Hopmans E. C. Pancost R. D. Sinninghe Damsté J. S. (2000). Widespread occurence of structurally diverse tetraether membrane lipids: evidence for the ubiquitous presence of low-temperature relatives of hyperthermophiles. Proc. Natl. Acad. Sci. U.S.A. 97, 14421–14426. 10.1073/pnas.97.26.14421 11121044 PMC18934

[B41] Schouten S. Van Der Meer M. T. J. Hopmans E. C. Rijpstra W. I. C. Reysenbach A.-L. Ward D. M.. (2007). Archaeal and bacterial glycerol dialkyl glycerol tetraether lipids in hot springs of Yellowstone National Park. Appl. Environ. Microbiol. 73, 6181–6191. 10.1128/AEM.00630-07 17693566 PMC2074994

[B42] Schouten S. Wakeham S. G. Hopmans E. C. Sinninghe Damsté J. S. (2003). Biogeochemical evidence that thermophilic archaea mediate the anaerobic oxidation of methane. Appl. Environ. Microbiol. 69, 1680–1686. 10.1128/AEM.69.3.1680-1686.2003 12620859 PMC150050

[B43] Shimada H. Nemoto N. Shida Y. Oshima T. Yamagishi A. (2008). Effects of pH and temperature on the composition of polar lipids in *Thermoplasma acidophilum* HO-62. J. Bacteriol. 190, 5404–5411. 10.1128/JB.00415-08 18539746 PMC2493274

[B44] Shock E. L. Holland M. E. (2007). Quantitative habitability. Astrobiology 7, 839–851. 10.1089/ast.2007.0137 18163866

[B45] Sinninghe Damsté J. S. Schouten S. Hopmans E. C. Van Duin A. C. T. Geenevasen J. A. (2002). Crenarchaeol: the characteristic core glycerol dibiphytanyl glycerol tetraether membrane lipid of cosmopolitan pelagic crenarchaeota. J. Lipid Res. 43, 1641–1651. 10.1194/jlr.M200148-JLR200 12364548

[B46] Sturt H. F. Summons R. E. Smith K. Elvert M. Hinrichs K. U. (2004). Intact polar membrane lipids in prokaryotes and sediments deciphered by high-performance liquid chromatography/electrospray ionization multistage mass spectrometry–new biomarkers for biogeochemistry and microbial ecology. Rapid Commun. Mass Spectrom. 18, 617–628. 10.1002/rcm.1378 15052572

[B47] Syakti A. D. Mazzella N. Torre F. Acquaviva M. Gilewicz M. Guiliano M.. (2006). Influence of growth phase on the phospholipidic fatty acid composition of two marine bacterial strains in pure and mixed cultures. Res. Microbiol. 157, 479–486. 10.1016/j.resmic.2005.11.001 16380233

[B48] Takano Y. Chikaraishi Y. Ogawa N. O. Nomaki H. Morono Y. Inagaki F. (2010). Sedimentary membrane lipids recycled by deep-sea benthic archaea. Nat. Geosci. 3, 858–861. 10.1038/ngeo983

[B49] Thompson D. H. Wong K. F. Humphry-Baker R. Wheeler J. J. Kim J.-M. Rannanavare S. B. (1992). Tetraether bolaform amphiphiles as models of archaebacterial membrane lipids: Raman spectroscopy,^31^P NMR, X-ray scattering, and electron microscopy. J. Am. Chem. Soc. 114, 9035–9042. 10.1021/ja00049a040

[B50] Tierney J. E. Schouten S. Pitcher A. Hopmans E. C. Sinninghe Damsté J. S. (2012). Core and intact polar glycerol dialkyl glycerol tetraethers (GDGTs) in Sand Pond, Warwick, Rhode Island (USA): insights into the origin of lacustrine GDGTs. Geochim. Cosmochim. Acta 77, 561–581. 10.1016/j.gca.2011.10.018

[B51] Uda I. Sugai A. Itoh Y. H. Itoh T. (2001). Variation in molecular species of polar lipids from *Thermoplasma acidophilum* depends on growth temperature. Lipids 36, 103–105. 10.1007/s11745-001-0914-2 11214723

[B52] Valentine D. L. (2007). Adaptations to energy stress dictate the ecology and evolution of the Archaea. Nat. Rev. Microbiol. 5, 316–323. 10.1038/nrmicro1619 17334387

[B53] Van De Vossenberg J. L. C. M. Driessen A. J. Zillig W. Konings W. N. (1998). Bioenergetics and cytoplasmic membrane stability of the extremely acidophilic, thermophilic archaeon *Picrophilus oshimae*. Extremophiles 2, 67–74. 10.1007/s007920050044 9672680

[B54] Veerkamp J. H. (1971). Fatty acid composition of *Bifidobacterium* and *Lactobacillus* strains. J. Bacteriol. 108, 861–867. 5128337 10.1128/jb.108.2.861-867.1971 PMC247153

[B55] Villanueva L. Damsté J. S. S. Schouten S. (2014). A re-evaluation of the archaeal membrane lipid biosynthetic pathway. Nat. Rev. Microbiol. 12, 438–448. 10.1038/nrmicro3260 24801941

[B56] Wei Y. Wang J. Liu J. Dong L. Li L. Wang H.. (2011). Spatial variations in archaeal lipids of surface water and core-top sediments in the South China Sea and their implications for paleoclimate studies. Appl. Environ. Microbiol. 77, 7479–7489. 10.1128/AEM.00580-11 21890672 PMC3209188

[B57] Xie W. Zhang C. Zhou X. Wang P. (2014). Salinity-dominated change in community structure and ecological function of Archaea from the lower Pearl River to coastal South China Sea. Appl. Microbiol. Biotechnol. 98, 7971–7982. 10.1007/s00253-014-5838-9 24880629

[B58] Yamauchi K. Doi K. Yoshida Y. Kinoshita M. (1993). Archaebacterial lipids: highly proton-impermeable membranes from 1,2-diphytanyl-sn-glycero-3-phosphocholine. Biochim. Biophys. Acta 1146, 178–182. 10.1016/0005-2736(93)90353-2 8383997

[B59] Yoshinaga M. Y. Gagen E. J. Wörmer L. Broda N. K. Meador T. B. Wendt J.. (2015). *Methanothermobacter thermautotrophicus* modulates its membrane lipids in response to hydrogen and nutrient availability. Front. Microbiol. 6:5. 10.3389/fmicb.2015.00005 25657645 PMC4302986

[B60] Zhang C. L. Wang J. Wei Y. Zhu C. Huang L. Dong H. (2011). Production of branched tetraether lipids in the lower Pearl River and estuary: effects of extraction methods and impact on bGDGT proxies. Front. Microbiol. 2:274. 10.3389/fmicb.2011.00274 22291686 PMC3253547

