# Supplementary material for: Influence of Growth Phase, pH, and Temperature on the Abundance and Composition of Tetraether Lipids in the Thermoacidophile Picrophilus torridus
Source: Front Microbiol. 2016 Aug 30;7:1323. doi: 10.3389/fmicb.2016.01323 (PMC5003844; doi:10.3389/fmicb.2016.01323)
Supplement: Supplementary file 1 [file DataSheet1.docx]

**Supplemental Online Material**

Influence of growth phase, pH, and temperature on the abundance and composition of tetraether lipids in the thermoacidophile *Picrophilus torridus*

Jayme Feyhl-Buska^1^, Yufei Chen^2^, Chengling Jia^2^, Jin-Xiang Wang^2^, Chuanlun L. Zhang^2^, and Eric S. Boyd^1,3*^

**Supp. Table 1.** Composition and abundance of GDGT and GTGT lipids in the core and polar fractions of *P. torridus* cells harvested at different points during its growth cycle. The average (Avg) and standard deviation (SD) of three replicate cultures is presented. The mass/charge ratio for each individual GDGT lipid is indicated with the number of cyclopentyl rings for that lipid in parentheses.

|  |  | **Relative Abundance** | | | | | | | | |  |  |  | |  |
| --- | --- | --- | --- | --- | --- | --- | --- | --- | --- | --- | --- | --- | --- | --- | --- |
| **Lipid Fraction** | **Growth  Phase** | **1286 (8)** | **1288 (7)** | **1290 (6)** | **1292 (5)** | **1294 (4)** | **1296 (3)** | **1298 (2)** | **1300 (1)** | **1302 (0)** | **GDGT (fg/cell)** | **GTGT (fg/cell)** | **Ring Index** |  |  |
| Core (Avg) | Lag | <0.01 | 0.11 | 0.93 | 9.55 | 39.39 | 12.68 | 24.48 | 4.69 | 8.18 | 16.7 | 0.4 | 3.03 |  |  |
|  | Early Log | <0.01 | 0.03 | 0.27 | 3.25 | 13.75 | 8.01 | 25.09 | 7.34 | 42.25 | 11.0 | 1.4 | 1.55 |  |  |
|  | Log | <0.01 | 0.10 | 0.63 | 6.00 | 18.64 | 9.73 | 27.83 | 8.57 | 28.49 | 2.2 | 0.3 | 2.03 |  |  |
|  | Late Log | <0.01 | 0.03 | 0.26 | 3.01 | 11.64 | 8.80 | 33.09 | 10.63 | 32.54 | 5.5 | 0.6 | 1.67 |  |  |
|  | Death | <0.01 | 0.05 | 0.49 | 7.20 | 23.84 | 15.75 | 34.66 | 8.76 | 9.25 | 35.6 | 1.0 | 2.60 |  |  |
| Core (SD) | Lag | <0.01 | 0.03 | 0.33 | 0.83 | 0.94 | 0.57 | 0.57 | 0.51 | 2.76 | 11.5 | 0.4 | 0.14 |  |  |
|  | Early Log | <0.01 | 0.02 | 0.22 | 2.35 | 8.62 | 5.02 | 11.46 | 1.04 | 28.72 | 4.0 | <0.1 | 0.88 |  |  |
|  | Log | <0.01 | 0.03 | 0.09 | 0.21 | 1.22 | 0.66 | 1.65 | 0.66 | 2.07 | 0.3 | <0.1 | 0.02 |  |  |
|  | Late Log | <0.01 | 0.01 | 0.10 | 0.28 | 0.54 | 0.61 | 1.29 | 1.00 | 0.19 | 1.0 | 0.2 | 0.01 |  |  |
|  | Death | <0.01 | 0.02 | 0.21 | 0.32 | 1.11 | 0.59 | 1.11 | 0.18 | 0.87 | 10.0 | 0.3 | 0.05 |  |  |
| Polar (Avg) | Lag | 0.01 | 0.06 | 0.74 | 7.75 | 34.73 | 12.84 | 30.32 | 5.90 | 7.64 | 16.3 | 0.6 | 2.90 |  |  |
|  | Early Log | <0.01 | 0.01 | 0.13 | 1.97 | 11.16 | 8.96 | 36.11 | 10.72 | 30.93 | 124.3 | 16.0 | 1.56 |  |  |
|  | Log | <0.01 | 0.04 | 0.32 | 3.34 | 13.83 | 11.78 | 37.83 | 11.45 | 21.41 | 38.3 | 3.6 | 1.89 |  |  |
|  | Late Log | <0.01 | 0.02 | 0.17 | 1.98 | 10.88 | 10.81 | 42.23 | 14.14 | 19.77 | 92.5 | 7.8 | 1.73 |  |  |
|  | Death | <0.01 | 0.02 | 0.22 | 5.58 | 22.57 | 16.08 | 38.42 | 9.73 | 7.39 | 89.6 | 2.8 | 2.50 |  |  |
| Polar (SD) | Lag | 0.01 | 0.03 | 0.07 | 1.23 | 3.87 | 1.55 | 2.50 | 1.19 | 0.18 | 0.3 | <0.1 | 0.10 |  |  |
|  | Early Log | <0.01 | <0.01 | 0.02 | 0.67 | 0.85 | 0.47 | 4.41 | 2.32 | 5.64 | 114.1 | 12.3 | 0.03 |  |  |
|  | Log | <0.01 | <0.01 | <0.01 | 0.13 | 0.49 | 0.61 | 0.70 | 1.33 | 2.02 | 34.4 | 3.0 | 0.01 |  |  |
|  | Late Log | <0.01 | <0.01 | <0.01 | 0.03 | 1.64 | 1.17 | 0.32 | 0.04 | 2.42 | 97.1 | 7.7 | 0.09 |  |  |
|  | Death | <0.01 | <0.01 | 0.04 | 0.60 | 0.24 | 1.49 | 0.40 | 0.11 | 1.86 | 72.6 | 1.7 | 0.11 |  |  |

**Supp. Table 2.** Composition and abundance GDGT and GTGT lipids in the core and polar fractions of *P. torridus* cells harvested during log phase growth at different cultivation medium pH. The average (Avg) and standard deviation (SD) of three replicate cultures is presented. The mass/charge ratio for each individual GDGT lipid is indicated with the number of cyclopentyl rings for that lipid in parentheses.

|  |  | **Relative Abundance** | | | | | | | | |  |  | |  |
| --- | --- | --- | --- | --- | --- | --- | --- | --- | --- | --- | --- | --- | --- | --- |
| **Lipid Fraction** | **Medium**  **pH** | **1286 (8)** | **1288 (7)** | **1290 (6)** | **1292 (5)** | **1294 (4)** | **1296 (3)** | **1298 (2)** | **1300 (1)** | **1302 (0)** | **GDGT (fg/cell)** | **GTGT (fg/cell)** | **Ring Index** | |
| Core (Avg) | 0.3 | 0.04 | 0.31 | 1.67 | 10.34 | 24.28 | 8.94 | 27.11 | 5.76 | 21.55 | 14.8 | 0.8 | 2.53 | |
|  | 0.5 | <0.01 | 0.08 | 0.54 | 4.93 | 16.57 | 7.15 | 21.15 | 6.08 | 43.49 | 4.2 | 0.7 | 1.63 | |
|  | 0.7 | 0.01 | 0.10 | 0.63 | 6.00 | 18.64 | 9.73 | 27.83 | 8.57 | 28.49 | 2.2 | 0.3 | 2.03 | |
|  | 0.9 | 0.01 | 0.10 | 0.56 | 4.60 | 14.32 | 6.42 | 19.76 | 6.29 | 47.94 | 1.1 | 0.4 | 1.48 | |
|  | 1.1 | <0.01 | 0.04 | 0.25 | 1.45 | 3.73 | 2.76 | 15.15 | 10.78 | 65.83 | 1.6 | 1.2 | 0.64 | |
| Core (SD) | 0.3 | <0.01 | 0.02 | 0.17 | 0.88 | 2.31 | 0.34 | 2.12 | 0.11 | 1.48 | 5.5 | 0.7 | 0.12 | |
|  | 0.5 | <0.01 | 0.07 | 0.46 | 4.65 | 15.59 | 5.88 | 8.67 | 0.27 | 35.05 | 2.9 | 0.4 | 1.27 | |
|  | 0.7 | <0.01 | 0.03 | 0.09 | 0.21 | 1.22 | 0.66 | 1.65 | 0.66 | 2.07 | 0.3 | 0.0 | 0.02 | |
|  | 0.9 | 0.01 | 0.08 | 0.38 | 3.11 | 9.10 | 3.37 | 1.16 | 0.76 | 16.44 | 0.1 | 0.2 | 0.68 | |
|  | 1.1 | <0.01 | 0.00 | 0.04 | 0.24 | 0.43 | 0.29 | 0.16 | 0.09 | 1.07 | 0.4 | 0.3 | 0.04 | |
| Polar (Avg) | 0.3 | 0.02 | 0.17 | 0.96 | 5.59 | 17.88 | 9.09 | 32.27 | 8.25 | 25.76 | 45.6 | 5.5 | 2.04 | |
|  | 0.5 | <0.01 | 0.05 | 0.45 | 5.13 | 22.98 | 14.97 | 36.33 | 7.85 | 12.23 | 72.0 | 3.8 | 2.43 | |
|  | 0.7 | <0.01 | 0.04 | 0.32 | 3.34 | 13.83 | 11.78 | 37.83 | 11.45 | 21.41 | 38.4 | 3.6 | 1.89 | |
|  | 0.9 | <0.01 | 0.02 | 0.16 | 1.91 | 9.12 | 7.22 | 36.24 | 10.93 | 34.39 | 32.3 | 4.4 | 1.43 | |
|  | 1.1 | <0.01 | 0.06 | 0.49 | 4.31 | 12.02 | 11.09 | 35.99 | 13.31 | 22.72 | 55.3 | 5.1 | 1.83 | |
| Polar (SD) | 0.3 | 0.01 | 0.04 | 0.21 | 1.17 | 2.90 | 0.76 | 0.31 | 0.37 | 4.41 | 19.1 | 2.4 | 0.22 | |
|  | 0.5 | <0.01 | 0.01 | 0.08 | 0.73 | 2.30 | 1.73 | 1.09 | 0.22 | 3.53 | 44.7 | 2.0 | 0.17 | |
|  | 0.7 | <0.01 | <0.01 | <0.01 | 0.13 | 0.49 | 0.61 | 0.70 | 1.33 | 2.02 | 34.5 | 3.0 | 0.01 | |
|  | 0.9 | <0.01 | 0.01 | 0.06 | 0.80 | 3.12 | 2.45 | 3.52 | 0.82 | 10.77 | 27.1 | 1.8 | 0.32 | |
|  | 1.1 | <0.01 | 0.02 | 0.17 | 1.54 | 3.37 | 2.92 | 1.11 | 0.65 | 8.49 | 31.1 | 3.2 | 0.35 | |

**Supp. Table 3.** Composition and abundance GDGT and GTGT lipids in the core and polar fractions of *P. torridus* cells harvested during log phase growth at different cultivation temperatures (°C). The average (Avg) and standard deviation (SD) of three replicate cultures is presented. The mass/charge ratio for each individual GDGT lipid is indicated with the number of cyclopentyl rings for that lipid in parentheses.

|  |  | **Relative Abundance** | | | | | | | | |  |  | |  | |  | |
| --- | --- | --- | --- | --- | --- | --- | --- | --- | --- | --- | --- | --- | --- | --- | --- | --- | --- |
|  | **Growth**  **Temp** | **1286 (8)** | **1288 (7)** | **1290 (6)** | **1292 (5)** | **1294 (4)** | **1296 (3)** | **1298 (2)** | **1300 (1)** | **1302 (0)** | **GDGT (fg/cell)** | | **GTGT (fg/cell)** | | **Ring Index** | |  |
| Core (Avg) | 53ᵒC | <0.01 | 0.08 | 0.73 | 6.39 | 13.34 | 5.69 | 22.97 | 6.60 | 44.19 | 7.3 | | 1.6 | | 1.60 | |  |
|  | 58ᵒC | 0.01 | 0.10 | 0.63 | 6.00 | 18.64 | 9.73 | 27.83 | 8.57 | 28.49 | 2.2 | | 0.3 | | 2.03 | |  |
|  | 63ᵒC | 0.01 | 0.20 | 1.13 | 8.13 | 18.18 | 9.39 | 23.92 | 7.62 | 31.42 | 2.6 | | 0.5 | | 2.05 | |  |
| Core (SD) | 53ᵒC | <0.01 | 0.01 | 0.11 | 0.97 | 2.21 | 0.62 | 1.68 | 0.18 | 5.79 | 1.3 | | 0.7 | | 0.21 | |  |
|  | 58ᵒC | <0.01 | 0.03 | 0.09 | 0.21 | 1.22 | 0.66 | 1.65 | 0.66 | 2.07 | 0.3 | | <0.1 | | 0.02 | |  |
|  | 63ᵒC | 0.01 | 0.20 | 1.13 | 8.13 | 18.18 | 9.39 | 23.92 | 7.62 | 31.42 | 1.4 | | 0.2 | | 0.26 | |  |
| Polar (Avg) | 53ᵒC | <0.01 | 0.05 | 0.49 | 4.53 | 11.18 | 8.01 | 36.34 | 10.91 | 28.48 | 102.1 | | 12.3 | | 1.72 | |  |
|  | 58ᵒC | <0.01 | 0.04 | 0.32 | 3.34 | 13.83 | 11.78 | 37.83 | 11.45 | 21.41 | 38.3 | | 3.6 | | 1.89 | |  |
|  | 63ᵒC | 0.01 | 0.08 | 0.55 | 4.64 | 17.16 | 13.26 | 39.72 | 11.08 | 13.51 | 40.3 | | 2.5 | | 2.20 | |  |
| Polar (SD) | 53ᵒC | <0.01 | <0.01 | 0.04 | 0.01 | 0.42 | 0.95 | 1.51 | 0.87 | 2.88 | 94.5 | | 3.4 | | 0.04 | |  |
|  | 58ᵒC | <0.01 | <0.01 | <0.01 | 0.13 | 0.49 | 0.61 | 0.70 | 1.33 | 2.02 | 34.4 | | 3.0 | | 0.01 | |  |
|  | 63ᵒC | <0.01 | 0.03 | 0.10 | 0.51 | 1.43 | 0.78 | 0.18 | 0.42 | 2.61 | 11.6 | | 1.6 | | 0.12 | |  |

**
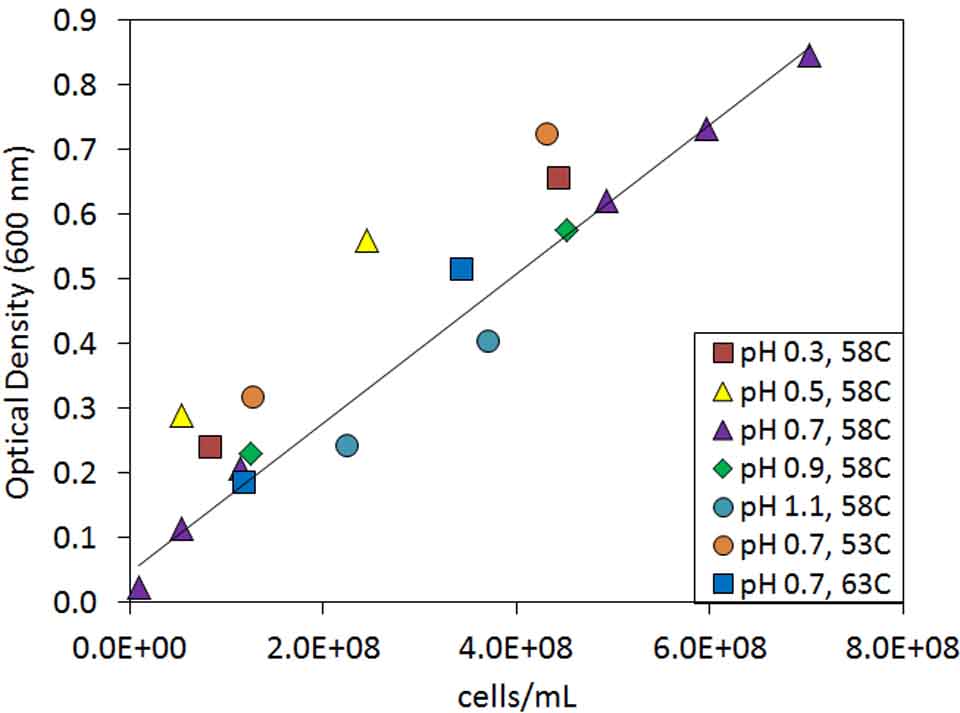
**

**Supp. Fig. 1.** Relationship between optical density (absorbance at 600 nm) and cell counts in cultures of *P. torridus* grown under various cultivation conditions, as specified in the legend.


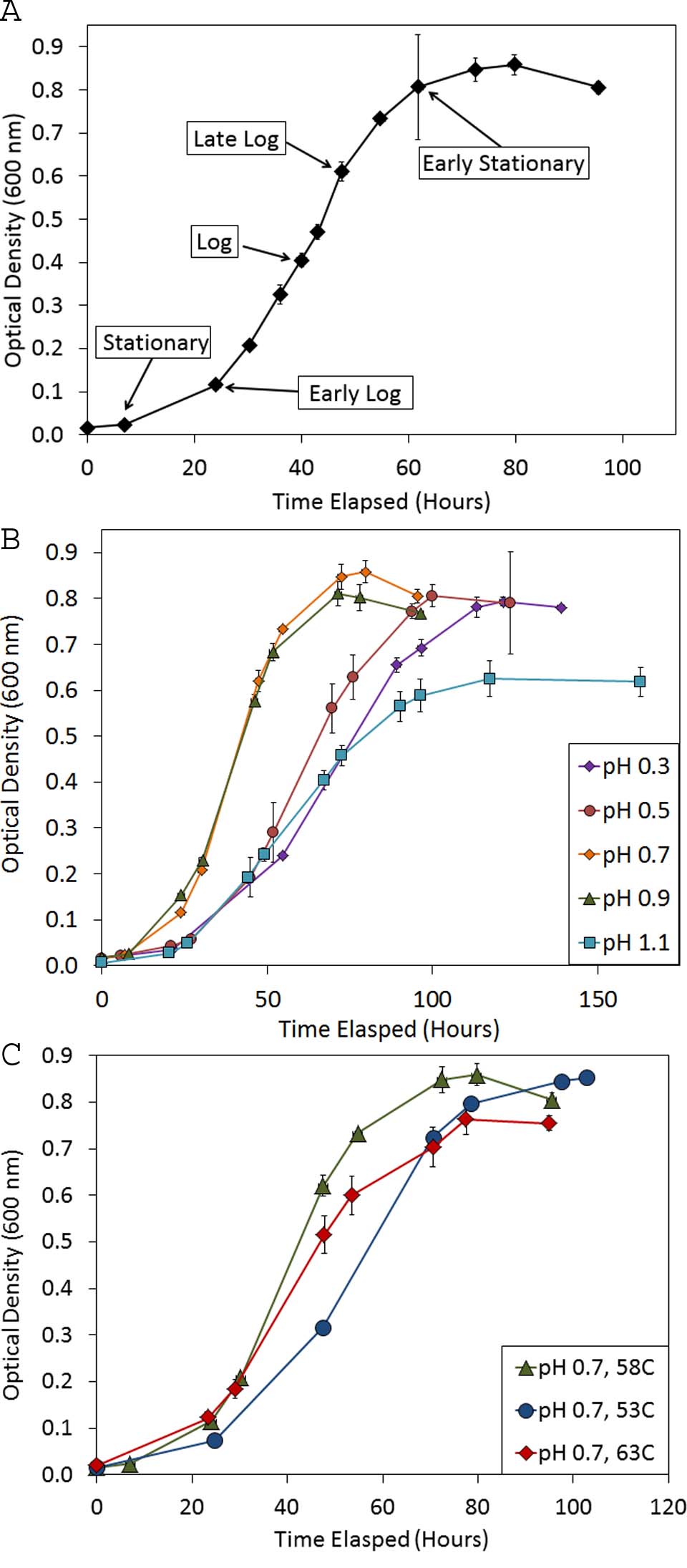


**Supp. Fig. 2.** Growth curves for cultures of *P. torridus* grown under optimal temperature (58⁰C) and pH (0.7) conditions with arrows denoting when samples were collected from each phase of growth (A). Growth curves for cultures of *P. torridus* grown under optimal temperature (58⁰C) and under varied pH conditions (B). Growth curves for cultures of *P. torridus* grown under optimal pH (0.7) and under varied temperature conditions (C).


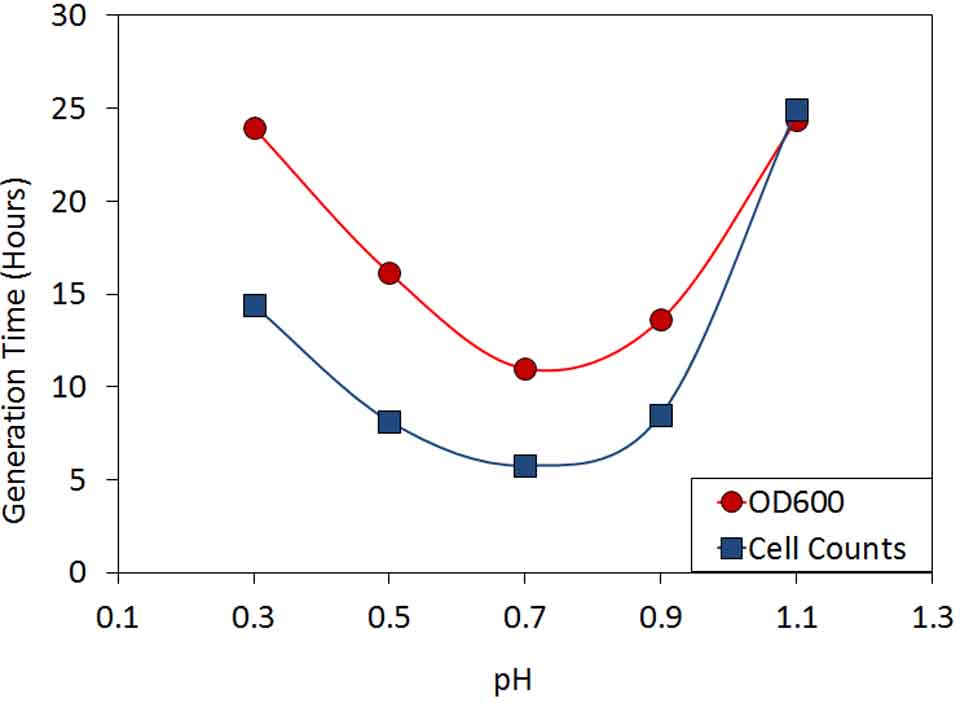


**Supp. Fig. 3.** Generation times for cultures of *P. torridus* as calculated using optical density (absorbance at 600 nm) or using direct cell counts.
